# Educational intervention for collecting sputum for tuberculosis: a quasi-experimental study**^1^**

**DOI:** 10.1590/1518-8345.0363.2703

**Published:** 2016-06-07

**Authors:** Amélia Nunes Sicsú, Julia Ignez Salem, Luciana Botinelly Mendonça Fujimoto, Roxana Isabel Cardozo Gonzales, Maria do Socorro de Lucena Cardoso, Pedro Fredemir Palha

**Affiliations:** 2 Doctoral student, Escola de Enfermagem de Ribeirão Preto, Universidade de São Paulo, PAHO/WHO Collaborating Centre for Nursing Research Development, Ribeirão Preto, SP, Brazil. Assistant Professor, Escola Superior de Ciências da Saúde, Universidade do Estado do Amazonas, Manaus, AM, Brasil. Scholarship holder from Fundação de Amparo à Pesquisa do Estado do Amazonas (FAPEAM), Brazil.; 3 Researcher, Instituto Nacional de Pesquisa da Amazônia, Manaus, AM, Brazil.; 4 Adjuntct Professor, Faculdade de Medicina, Universidade Federal do Amazonas, Manaus, AM, Brazil.; 5 Adjunct Professor, Faculdade de Enfermagem e Obstetrícia, Universidade Federal de Pelotas, Pelotas, RS, Brazil.; 6 Associate Professor, Escola de Enfermagem de Ribeirão Preto, Universidade de São Paulo,

**Keywords:** Tuberculosis, Health Education, Specimen Handling, Intervention Studies

## Abstract

**Objective:**

to evaluate the quality of the sputum sample before and after the Nursing guidance to patients.

**Methods:**

this is a quasi-experimental research design, single group type, before and after, non-randomized study. The study enrolled patients with suspected pulmonary tuberculosis, respiratory symptomatic patients for over three weeks, aged over 18 years, of both genders and without tuberculosis history in the last two years. The educational intervention consisted of individualized guidance on the collection of sputum sample, which was based on the guidelines of the Ministry of Health of Brazil and on the explanatory folder delivery.

**Results:**

in this study participated 138 patients with suspected pulmonary tuberculosis. The results showed significant increase of the samples with purulent particles, volume greater than 5 mL and increased rate of patients diagnosed with tuberculosis, after the educational intervention.

**Conclusion:**

it was shown that after the educational intervention, it was observed sputum samples with better quality, with satisfactory aspect and volume for the effectiveness of the bacilloscopic examination.

## Introduction

The early diagnosis is one of the priority strategies for the control of tuberculosis (TB), a disease that remains a serious public health problem of worldwide magnitude. Of the 5.4 million new cases reported in 2013, over 80% presented the pulmonary form of the disease^1^.

The diagnostic confirmation is based on the clinical and epidemiological history, radiological examinations, tuberculin skin test, bacteriological tests (bacilloscopy and culture), and biochemical and molecular tests^2^ **_._** Although the expectation of expansion of the rapid test for detecting TB is high for all global health services, bacilloscopy remains the priority method, especially in developing countries, because it is simple, fast, inexpensive and, when properly executed, it allows to identify about 70% of cases of pulmonary TB^3^.

It is worth mentioning that regardless of the technological innovations introduced to diagnosis, the quality of sputum sample plays a central role, since the accuracy of any laboratory results of TB diagnosis depends on the quality of a collected sample^4^. Accordingly, the efficiency in the detection of new TB cases is intrinsically related to the quality of the sample and this, in turn, depends on the quality of the guidance on how to properly collect the material.

The good professional/patient interaction, acceptability and understanding of the guidelines provided by the health professionals influence the quality of the sample, avoiding the presence of saliva and nasopharyngeal secretions, which are not appropriate for laboratory examinations^5^. The guidelines should be carried out in a clear fashion, respecting the heterogeneity and uniqueness of each individual's learning. Qualifying guidelines can reduce the universe of patients with TB and undiagnosed patients due to the poor quality of the collected sample, which remain for long periods spreading the disease and hampering the efficiency/effectiveness of the actions of the Tuberculosis Control Programme.

In the context of TB care, the nurse has assumed an important role in the prevention and control of the disease, which ranges from actions such as identification of respiratory symptoms to discharge of the confirmed cases^6^. In this sense, the clinical nursing practice, supported by the Evidence-Based Practice (EBP), is of fundamental importance since it guides the management and care decision process of Nursing^7^. This, in turn, is based on problem identification, consensus of the relevant scientific evidence, implementation of these in professional practice and evaluation of the results achieved^8^, a process which should be in line with the preferences of the patient. Thus, the use of EBP represents a qualifier for the health care at different levels of care, resulting in a better clinical practice^7^.

This study is justified by the few evidence found^9^ ^-^ ^14^, after a bibliographical survey in LILACS, PubMed, CINHAHL, Web of Science and Scopus databases, on the importance of education in health, aiming at the quality of the sputum sample and improvement of diagnosis.

Over the past five years, it was found only one study carried out in Brazil, addressing the impact of guidance for sputum sample collection, however, its design was focused on the evaluation of the culture contamination rate and not on the direct impact of the guidelines regarding the quality (aspect and volume) of the sample and bacilloscopic results^14^ **_._**

It is relevant to carry out studies, whose results reinforce the scientific evidence on the effectiveness of the guidance on the appropriate sputum sample collection. It is highlighted that this is the first national study to assess the contribution of the health education to patients, for the adequate collection of sputum samples, aiming at contributing to the improvement of the health service diagnostic capability.

Accordingly, this study aimed to evaluate the quality of the sputum sample for the laboratory diagnosis of TB, before and after Nursing guidelines in symptomatic respiratory patients with suspected TB.

## Method

This is a quasi-experimental research design, single group type, before and after, non-randomized study, approved by the Committee of Ethics and Research on Humans of the Federal University of Amazonas (CEP/UFAM), under Protocol CAE number 0001.0.113.115-10. In this type of study, the patient is his own control, before and after intervention. Therefore, the following variables were compared: aspect, volume and bacilloscopic results of the sample before and after the educational intervention.

The study was carried out in the Reference Center for Sanitary Pneumology (CREPS) "Cardoso Fontes". This site was chosen because it was and still is responsible for diagnosing 70% of patients with TB in the state of Amazonas, Brazil, although the process of decentralization of diagnosis and treatment of TB for primary care occurred from 2003.

The cases diagnosed in this reference unit are referred for continuity of care and reporting on the Basic Health Unit closest to the residence of the patient^15^.

Usually the unit assists, on average, from 900 to 1000 patients per month (respiratory symptomatic patients of all age groups and presenting newly reported or recurrent symptoms for more than a week), of which approximately 7-8% have pulmonary tuberculosis with positive bacilloscopy, which were referred for routine medical monitoring and treatment of disease.

The study population consisted of respiratory symptomatic patients with suspected pulmonary TB, who sought care at CREPS "Cardoso Fontes", from September 2010 to February 2011.

The selection of patients took place on alternate days, because of the need of receiving the sputum sample on the day after the identification of the respiratory symptoms. Participants met the following inclusion criteria: patients with suspected pulmonary TB, respiratory symptomatic for more than three weeks, over 18 years old, of both genders, without TB history in the last two years, which performed and/or provided the third sputum sample on the scheduled day.

All patients who met the inclusion criteria were invited to participate in the study at the time of delivery of the second sample in the health service. Of the total of participants who met the inclusion criteria (270 patients), 85.2% (230 patients) agreed to participate in the study upon acceptance of the Informed Consent Form (IC). On average, 20 patients per day met the inclusion criteria.

Upon acceptance for the IC, the researcher nurse referred these patients to a specific room next to the laboratory to receive guidance on the collection of the sample of sputum individually. Only 138 patients provided the third sputum sample on the scheduled day, totaling thus the amount of study participants. Thus, the intentional non-probabilistic sample of participants was composed of 138 patients.

Patients underwent the collection of three sputum samples. The first was carried out in the health service, the second, on the following day, at the residence of the patient and the third at home, on the day after the delivery of the second sample. It is noteworthy that the first and second samples were collected according to the guidance provided by the health professionals of the CREPS "Cardoso Sources" and processed only for the bacilloscopic examination.

The collection of the third sample was carried out after the educational intervention by the nurse researcher and it consisted of discussing, individually, the guidelines recommended by the Ministry of Health^16^.

The intervention was based on the guidelines of the Ministry of Health of Brazil (2008). The researcher nurse carried out all steps. The guidelines were given in an office appropriated for the intervention, made available by the coordinator of the local. The duration of the intervention was between 15 to 20 minutes and included the following aspects^16^: explanation on the importance of the test to patient, using easy-to-understand language; guidance on the importance of following the steps of the collection procedure in order to collect material from the bronchial tree and not from the oropharynx, since saliva samples are inappropriate for bacteriological analysis, as it does not represent the infectious process; advice to drink plenty of fluids in the day before the sputum collection, at least eight glasses, due to the need to fluidize the secretions adhered to the bronchial wall. The patient must sleep, preferably in a horizontal position and without a pillow to facilitate the release of sputum at the time of collection, guidance for tooth brushing, and making mouthwash with water before sputum collection. In fasting, the patient must go alone to a well-ventilated place, outdoors and perform the procedures related to the proper handling of the supplied sputum collection vial and sequentially, to perform the breathing technique to cause cough and release of the sputum, considering the biosecurity measures related to packaging of the material and hand hygiene. These procedures are presented in detail in the National Manual of the Surveillance Laboratory for Tuberculosis and other Mycobacteria^16^. Finally, the patient was instructed to take the vial to the room set to receive the material collected at the CREPS "Cardoso Fontes", on the same day of the collection, since this material should not be stored at home and needs to be immediately processed for accurate test results.

After the educational intervention, the patients were asked to describe the steps required to carry out the collection, aiming at evaluating their understanding about the information provided. This step was important to detect gaps in the understanding on the guidelines and provide clarifications in case of doubt.

Subsequently, a transparent collection vial and an explanatory folder was given to all patients. The folder has a visual content with illustrations focused on the main stages of the collection procedure. It was used to facilitate the understanding of patients at the time of presentation of information and so that they could remember the steps when performing the collection at home. It is routinely used in the laboratory of the unit of Mycobacteriology of the National Institute of Amazonian Research (INPA) and was designed by its team of researchers.

The third sample was delivered to the researcher at the CREPS "Cardoso Sources". At the time of delivery carried out by the patient, the volume and aspect of the sample were evaluated.

In case of unsatisfactory samples, the patients were instructed again and a new attempt of collection was performed by the patients in their own health services, in an outside area, destined to this activity. If the new sputum collection was satisfactory, it was considered for analysis. The samples were processed in the Mycobacteriology Laboratory of INPA, through bacteriological techniques using direct microscopy, bacilloscopy after concentration and cultivation, routinely performed on the sputum samples received, regardless of this study.

To collect the data, two forms were used: one containing items on the sociodemographic and clinical characteristics of patients and the other one for registration of the aspect, quantity and the bacilloscopic score corresponding to the three samples of sputum collected. The classification criteria were made as recommended by the Ministry of Health^16^. The aspect of the sample was classified as saliva, liquefied, mucous, mucopurulent and bloodstained. The volume was classified as equal to or greater than 5 mL and lower than 5mL. The bacilloscopic results were classified into negative, inconclusive, a cross symbol, two crosses and three crosses.

The analysis and presentation of data were performed according to the research objectives. The results of the patient profiles were analyzed by means of statistical measures using absolute frequency and percentage.

The effect of the intervention was evaluated by comparing the second with the third sample collected by the study participants, in relation to three variables: aspect, volume and bacilloscopic results.

Regarding the aspect, samples were reclassified as inappropriate and appropriate according to the presence or absence of purulent particles. Regarding the volume, the parameters used were equal or greater than 5 mL (appropriate) and less than 5mL (inappropriate). To analyze the bacilloscopic results, it was established a re-categorization as negative and positive (+, ++, +++)^16^.

To evaluate the effect of the intervention on the aspect and volume of the sample and on the bacilloscopic results, it was used the McNemar test. The level of significance of the statistical test was defined as 5% (alpha=0.05).

## Results

Of the 138 respiratory symptomatic participants of this study, most were female (50.7%), mean age of 49.7, standard deviation=SD=15.7 years, income from 1 to 5 minimum wages (55.1%), slept in bed (71.0%), had BCG vaccination history (70.3%), did not perform tuberculin skin test (73.2%) and had a family history of TB (63.8%). The predominant category for marital status was married (43.5%) and incomplete elementary education (43.5%) as education level.

Regarding the results after the intervention, the results (Table 1) show that there was an increase of 10.2% of samples with purulent particles, 10.1% presented volume greater than 5 mL and improvement of 13.0% in the bacilloscopic diagnostic. 

**Table 1:** Distribution of sputum samples according to the variables: aspect, volume and bacilloscopic results, before and after intervention. Manaus, AM, Brazil, 2011

Variable		Before intervention (n=138)				After intervention (n=138)		McNemar test
		n	%		n	%	
Aspect							0.016
	Appropriate	86	62.3		100	72.5	
	Inappropriate	52	37.7		38	27.5	
Volume							0.007
	≥5mL	93	67.4		107	77.5	
	<5mL	45	32.6		31	22.5	
Bacilloscopy results							>0.05
	Positive	17	12.3		20	14.5	
	Negative	121	87.7		118	85.5	

It is observed that, in terms of aspect and volume of the sample, patients after the educational intervention were able to produce more samples with purulent particles and satisfactory volumes than before the intervention. This association was statistically significant. Regarding the bacilloscopic results, it was observed an improvement in the bacilloscopic diagnosis. However, these results were not statistically significant.

The evolution in the aspect of sputum samples before and after intervention is presented in Table 2.

**Table 2:** Distribution of the aspect of the sputum samples before and after intervention. Manaus, AM, Brazil, 2011

Sputum aspect	Before intervention (n=138)			After intervention (n=138)	
	n	(%)		n	(%)
Saliva	20	14.5		04	2.9
Liquefied	06	4.3		04	2.9
Mucous	20	14.5		21	15.2
mucopurulent	86	62.3		100	72.5
Bloodstained	06	4.3		09	6.5

After the intervention, the samples with aspect of saliva decreased and consequently, the frequency of the samples with mucopurulent aspect increased 10.2%.

Of the 138 respiratory symptomatic patients, 23 (16.7%) were diagnosed with pulmonary TB by means of the isolation procedure for *M. tuberculosis.* In Table 3, it is observed the proportion of cases diagnosed per each sputum collection stage.

**Table 3:** Absolute frequency and percentage of the bacilloscopy results, obtained for the 3 samples in the 23 cases of pulmonary tuberculosis, confirmed by examination of cultivation. Manaus, AM, Brazil, 2011

Bacilloscopy results	Pulmonary tuberculosis cases confirmed by cultivation (n=23)				
	Negative for AARB*				Positive for AARB*
	n	%		n	%
1^st^ sample	11	47.8		12	52.2
2^nd^ sample	5	21.7		17	73.9
3^rd^ sample	3	13.0		20	87.0

*Acid-Alcohol Resistant Bacilli

The first sample collected in the health service accounted for 52.2% of the positive bacilloscopy cases. The increase observed for the second sample corresponded to 21.7% and, after the intervention (3rd sample), it was identified increase of 13.0%.

## Discussion

The evaluation of the quality of the sputum samples for TB diagnosis examination before and after the Nursing instructions to patients, showed that intervention resulted in samples with better aspect, greater volumes and increased rates of patients diagnosed with TB.

The decrease in the percentage of samples with aspect of saliva, after the intervention, was also observed in another study^12^ **_._** If the sample is inappropriate (that is, only saliva), it may not be possible to find bacilli, even though the patient is bacillipherous^6^. Thus, it is evident that after the qualified instructions, as recommended, patient is able to reduce the number of samples with aspect of saliva, which consequently reduces the probability of false negatives.

Other studies have shown the positive effect of guidelines on the quality of the sample^9^ ^,^ ^11^ ^-^ ^13^. It was identified a positivity rate from 12.0 to 15.0% higher among patients who properly received the recommended guidelines by means of protocols^9^ ^,^ ^12^.

The sensitivity of the bacilloscopy test for pulmonary TB showed significant improvement when the sample volume was equal or higher than 5mL^12^. However, both the aspect and volume are important so that diagnostic tests for pulmonary TB present improved effectiveness.

Although the relationship between the bacilloscopic results and the intervention did not shown statistically significant difference, it is important to emphasize that, as shown in Table 3, there was a significant difference from the clinical point of view, since a single undiagnosed case has epidemiological importance on TB control and it contributes to the perpetuation of the disease transmission chain.

As shown in a recent study^17^, it is assumed that, within one year, a diseased or a bacillipherous individual can infect around 10-15 people with whom they maintained contact, because while they do not start treatment, they will continue to spread bacteria to the rest of the population. Once infected, about 5 to 10% of people develop the disease, half of them during the first two years after infection, and half of them later, depending on the immune conditions of the host and the reactivation of the bacilli from their latent state.

It is emphasized the importance of sputum collection usually carried out in two different locations. According to the program rules, the first is performed in the health service and represents an important contribution to the initial screening of cases. The second is usually performed at home, as it raises the identification potential due to the possibility of sputum collection at better physiological conditions (greater amount of material produced during the nocturnal rest) and at better safety conditions of the individual, since this procedure is performed at home. In recent studies, it was found that collection performed in the morning leads to improved efficiency in detecting AARB than the local collection^18^ ^-^ ^19^.

Although it was identified a percentage of 52.2% of positive bacilloscopy cases in the sample collected in the health service, the results could have been more effective regarding the diagnosis of TB, if a qualified guidance was provided, as proposed in this study. However, some guidelines are specific to the collection of the second sample (intake of a large amount of fluid on the day before collection, sleep without pillow and in a horizontal position, and tooth brushing before the collection), since this is performed at home. In this study, the educational intervention was effective to 13% of patients, which presented negative bacilloscopy results in the first two samples collected from biological material.

It is worth mentioning that 78.3% of cases of pulmonary TB were diagnosed at CREPS "Cardoso Fontes" by means of direct bacilloscopy (1st and 2nd sample). It is estimated that 70-80% of the estimated number of active bacilliferous cases are identified by means of bacilloscopy, indicating that the local service is achieving the diagnostic goals^3^. However, given the infectivity of the disease, health services should be concerned with not only meet the goals set by the National TB Control Programme, but also continually search for more comprehensive results, which can be obtained by providing educational interventions, according to the norms of the program.

It is worth emphasizing that interventions such as those used in this study are inexpensive and easily replicable in different contexts, since it is necessary only a place reserved and intended for carrying out the guidelines, a trained professional and the distribution of an explanatory folder. It is believed that delivery of the explanatory material contributes to assimilate the information, since it is a way for the patient to remember the steps necessary to perform the collection at home. In addition, it is important that professionals incorporate the strategy of checking the volume and aspect of the sample at the time of its delivery by the patient at the health service and, if the sample is unsatisfactory, a new qualified guidance should be provided for collecting a new sample, thus favoring the quality of the sputum collection.

A possible bias of the study is related to the guidance provided by the health professionals to patients about the collection of the previous samples. However, it was found by non-participant observation, that these guidelines did not follow the protocols recommended by the Ministry of Health and did not take into account the patients' heterogeneity. This reinforces the hypothesis that improvement in the quality, quantity and bacilloscopy results of the third sample is due to the intervention proposed in this study.

## Conclusion

The study showed that, after the educational intervention, according to the protocol established by the Ministry of Health of Brazil, it was obtained sputum samples with higher quality, with satisfactory aspect and volume for a better effectiveness of the bacilloscopic examination. It was identified an increase of 13% in the diagnosis of cases of bacilloscopic positive compared to the two previous collections.

It is evidenced, therefore, that educational interventions provide greater effectiveness in diagnosing the disease, reduction of false negative bacteriological results and strong potential in reducing the transmissibility of the disease.

Accordingly, there is a need of raising awareness among health professionals, of proper environments (good ventilation and away from the flow of people) and preparation of educational mechanisms that allow the transfer of all necessary guidelines for collecting sputum, using communication techniques that favor the understanding and incorporation of knowledge by people with different educational levels and heterogeneous life contexts.

## References

[1] World Health Organization (2014). Global tuberculosis report 2014.

[2] Bento J, Silva AS, Rodrigues F, Duarte R (2011). Métodos diagnósticos em tuberculose. Acta Med Port.

[3] Reed SL, Mamo G, Gossa E, Jasura M, Getahun M, Lemma E (2011). Improved tuberculosis smear detection in resource-limited settings
Combined bleach concentration and LED fluorescence microscopy. Int Health.

[4] Pinto LM, Udwadia ZF (2013). Xpert MTB/RIF and pulmonary tuberculosis time to delve
deeper?. Thorax.

[5] Hadad DJ, David AP, Brum DL, Nogueira LR, Sales CMM, Fregona G (2014). Metodologia para coleta de escarro espontâneo para confirmação
microbiológica do diagnóstico de tuberculose pulmonar, doença pulmonar por
micobactérias não tuberculosas ou para controle de tratamento desses agravos em
ambientes ambulatorial e hospitalar. J Infect Control.

[6] Oblitas FYM, Loncharich N, Salazar ME, David HML, Silva I, Velásquez D (2010). Nursing's role in tuberculosis control a discussion from the
perspective of equity. Rev. Latino-Am. Enfermagem.

[7] Spiri WC, MacPhee M (2013). The Meaning of Evidence-Based Management to Brazilian
Senior. J Nurs.

[8] Hung H, Huang YU, Tsai J, Chang Y. (2015). Current state of evidence-based practice education for undergraduate
nursing students in Taiwan: A questionnaire study.. Nurse Educ Today.

[9] Alisjahbana B, Van Crevel R, Danusantoso H, Gartinah T, Soemantri ES, Nelwan RHH (2005). Better patient instruction for sputum sampling can improve microscopic
tuberculosis diagnosis. Int J Tuberc Lung Dis.

[10] Macq J, Solis A, Velázquez A, Dujardin B (2005). Informing the TB suspect for sputum sample collection and
communicating laboratory results in Nicaragua a neglected process in tuberculosis
case finding. Salud Pública México.

[11] Alisjahbana B (2007). Improved diagnosis of tuberculosis by better sputum
quality. Thelancet.

[12] Khan MS, Dar O, Sismanidis C, Shah K, Godfrey-Faussett P (2007). Improvement of tuberculosis case detection and reduction of
discrepancies between men and women by simple sputum-submission instructions a
pragmatic randomized controlled trial. Lancet.

[13] Gonzalez AV, Menzies D (2008). In women with suspected TB, brief sputum-submission instruction
improved sampling quality and TB detection. EBM.

[14] Maciel ELN, Prado TN, Peres RL, Palaci M, Johnson JL, Dietze R (2009). Associação entre coleta de escarro guiada e taxas de contaminação de
culturas para o diagnóstico de TB pulmonar. J Bras Pneumol.

[15] Braga JU, Pinheiro JS, Matsuda JS, Barreto JAP, Feijão AMM (2012). Fatores associados ao abandono do tratamento da tuberculose nos
serviços de atenção básica em dois municípios brasileiros, Manaus e Fortaleza,
2006 a 2008. Cad Saúde Coletiva.

[16] Ministério da Saúde. Secretaria de Vigilância em Saúde. Departamento de
Vigilância (2008). Manual Nacional de vigilância laboratorial da Tuberculose e outras
micobactérias.

[17] Ferraz AF, Valente JG (2014). Epidemiological aspects of pulmonar tuberculosis in Mato Grosso do
Sul, Brazil. Rev Bras Epidemiol.

[18] Abraham PR, Sharma VD, Shivannavar CT (2012). Diagnosis of TB from smear & culture negative sputum specimens by
IS 6110 based PCR. Indian J Med Res.

[19] Islam MR, Khatun R, Uddin MKM, Khan SR, Rahman T, Ahmed T (2013). Yield of Two Consecutive Sputum Specimens for the Effective Diagnosis
of Pulmonary Tuberculosis. Plos One.

